# Temporary Teeth

**Published:** 1885-04

**Authors:** E. J. Lilly


ARTICLE V.

### TEMPORARY TEETH.

E. J. LILLY, M. D., D. D. S.

Several days ago a father brought to our office his seven-year-old son, suffering from a badly diseased temporary tooth. The father was one of those fussy, fidgety men, always making some remark, but never saying the right thing at the right time. The child was almost scared to death, and when he discovered he was at "the dentist's,"

seemed to about to collapse. He had been systematically deceived in regard to the cruelty and "horridness" of a dental operation, and was consequently wrought up to a pitch of fear and dread, that ought to have put the father to shame. We tolerated the man's talk for a few minutes, then told him very pleasantly, but firmly, that we would take the boy into another room, and ascertain what could be done for him. After securing his confidence by asking a few questions, and telling him a short story, we examined his tooth and told him what was necessary to be done in order to relieve him. He was then ready to have his tooth treated. This was accomplished without any more ado, much to the surprise and gratification of the father. We then explained, as well as our limited time allowed, the benefits of early consulting a dentist, and he promised not to be so negligent in the future.

In dealing with children, the dentist must be kind and considerate, but firm, and have a strict regard for the truth. We must remember they will be our future patients. Untruthful statements, rough manipulation, and a severe demeanor, leave the impression on the mind that the "horrid dentist" is a person to be avoided. If we do not make it as pleasant as possible for them when young, in after life they will apply to us only as the last resort.

We presume most dentists have found that operating for children is difficult and tedious, and often requires great patience and self-sacrifice. But after gaining their confidence, and by gentle manipulation, the operation can be successfully performed. But it must be remembered in operating that the pulps of these teeth are relatively large, and come near the surface. The work, nevertheless, should be thorough, as their texture renders inperfect fillings almost valueless. * * * * * *

In general, the deciduous teeth have received attention only when the sufferings of the child have rendered palliative treatment necessary, preventive treatment being comparatively a rare procedure. Cleanliness is as essential here as with the second set. The first step should be to teach the use of the brush, and, when necessary, the removal of the stain or deposit which often adheres with tenacity to the enamel. The use of the brush should be taught as soon as the child has sufficient intelligence to learn it; and previous to that time, this duty should be done for him. They should be brushed three times a day with a soft brush, on which has been placed a little soap and powder, brushing up, down and across.

These small teeth accomplish admirably the purpose for which they were designed, and it is our duty when called on for advice to expend the energy, exercise the patience and tolerate the annoyances necessary to keep them in good condition till they give place to the more permanent denture. There is a common belief among parents that the "first teeth" are unworthy of any attention except that given by the forceps. But they have a useful purpose to subserve, and are really indispensible to the health of the child. Besides they bear such intimate relations to the permanent teeth as to render their preservation of vital importance.

The temporary differ from adult teeth in size, form and dentisty, and require to be carefully dealt with till the child is old enough to do without them. Between the fourth and ninth year is the period demanding the most attention, the child at this time being sufficiently manageable A pleasing story, or a talk about their plays or pastimes will most always gain their good-will, and this is half the battle. Parents must be advised to have their children's teeth examined at intervals, and the importance of this should be impressed on their minds. It is not enough to urge and persuade them to do this; we must, rather, make clear to their understandings the reason why one thing is right and another wrong. Mothers, specially, have a great tenderness for their offspring; for this reason it is easy to find an excuse for neglect. That they should be anxious about them at this time is perfectly natural; but that the solicitude should be carried so far as to deprive the child of our care is not wise. If parents would feel the weight of responsibility under which they are placed, and act on it, their children would reap the benefits of greater care and attention than many of them receive. If they were made to understand how much pain and discomfort could be prevented and spared their little ones, for whom they would sacrifice anything, the long and wakeful nights they are sometimes compelled to pass, the money to be expended in regulating and filling, and the many other troubles that might be avoided by timely care and attention, we are sure they would thoroughly wake to their duty. Decayed, aching and dead teeth are, oftener than many imagine, the prime cause of weak eyes, ear-troubles, and headache; they may even cause deafness, blindness and other ills. Then let us endeavor to impress the important truth that prevention is better than cure.—[*Ohio State Journal.*]

---

ARTICLE VI.

